# *Sarothrogammarusyiiruae*, a new species of Amphipoda (Gammaridae) from China

**DOI:** 10.3897/zookeys.861.35538

**Published:** 2019-07-08

**Authors:** Yami Zheng, Zhonge Hou, Shuqiang Li

**Affiliations:** 1 Key Laboratory of Zoological Systematics and Evolution, Institute of Zoology, Chinese Academy of Sciences, Beijing 100101, China Institute of Zoology, Chinese Academy of Sciences Beijing China; 2 University of Chinese Academy of Sciences, Beijing 100049, China University of Chinese Academy of Sciences Beijing China

**Keywords:** genetic distance, sarothrogammarid amphipods, taxonomy, Tethys

## Abstract

A relic amphipod of the Tethys, *Sarothrogammarusyiiruae***sp. nov.**, is described from Xinjiang, China. The new species is characterized by the absence of eyes; having the palm of the propodus without a mid-palmar spine on gnathopods I–II; a weakly concave coxal plate IV; narrow bases of pereopods V–VII; a peduncle of uropod I without a basofacial spine; uropod III longer than uropods I–II, a scale-like inner ramus, and a biarticulate outer ramus with distinct second article. Detailed morphological comparisons with related species are discussed. Genetic distances of the new and related species are provided as proof of species identification.

## Introduction

Sarothrogammarid amphipods are considered relics of the Tethys fauna (Stock 1971, 1995), consisting of a freshwater *Sarothrogammarus*-group in Pamir and a brackish genus, *Rhipidogammarus* Stock, 1971 along the Mediterranean coast (Hou et al. 2011). This disjunct distribution is related to the Late Eocene retreat of the Tethys from Pamir (Hou et al. 2014). The freshwater *Sarothrogammarus*-group is characterized by having filtrative setae on pereopod III and urosomites with reduced spines or setae. The group contains the genera *Sarothrogammarus* Martynov, 1935, *Comatogammarus* Stock, 1971, and *Barnardiorum* Iwan & Löbl, 2007 (=*Tadzhikistania* Barnard & Barnard, 1983). The genus *Comatogammarus* is distinguished from *Sarothrogammarus* in having filtrative setae on pereopod IV; while the genus *Barnardiorum* differs from *Sarothrogammarus* in having a vestigial second article on uropod III. Up to now, there are eight species recorded in Pamir: *Comatogammarusferghanensis* (Martynov & Behning, 1948), *Barnardiorumruffoi* (Karaman, 1971), *B.shadini* (Birstein, 1948), *Sarothrogammarusasiaticus* Martynov, 1935, *S.multipennatus* Karaman, 1969, *S.lindbergi* Karaman, 1969, *S.afghanus* (Ruffo, 1958), and *S.trichiatus* Stock, 1971. They were all reported from Afghanistan or Tadzhikistan.

To explore the retreat route of the Tethys, an expedition was organized along the south side of the Tian Shan, China in 2014. A new species of *Sarothrogammarusyiiruae* sp. nov. was found from the east margin of Pamir. In the current paper, the new species is described and illustrated. The genetic distances between the new species and known species are calculated to confirm the species delimitation.

## Materials and methods

### Morphological observations

The specimens were collected with a fine-meshed hand net. Samples were preserved in 95% ethanol in the field and deposited in a -20 °C refrigerator for long term preservation. The body length was recorded by holding the specimen straight and measuring the distance along the dorsal side of the body from the base of the first antenna to the base of the telson. All dissected appendages were mounted on slides and were drawn using a Leica DM2500 compound microscope equipped with a drawing tube. Terminology and taxonomic descriptions follow the literature (Zheng and Hou 2017). The terms ‘‘setae’’ and ‘‘spines’’ are used to distinguish between thin or fine and more robust setal structures. All types and other materials are lodged in the Institute of Zoology, Chinese Academy of Sciences (IZCAS), Beijing.

### Molecular methods

Partial fragments of mitochondrial cytochrome oxidase subunit (COI) and nuclear 28S rRNA were amplified to confirm identifications. Genomic DNA extraction, amplification and sequencing procedures were performed as in Hou et al. (2007). Uncorrected pairwise distances were calculated using MEGA 7.0.16 (Kumar et al. 2016). The new sequences were deposited in GenBank and the accession numbers are provided in Tables 1, 2.

**Table 1.** GenBank accession numbers and uncorrected pairwise distances of the COI partial sequences between species in this text.

	Species	Genbank accession number	1	2	3	4
1	* Comatogammarus ferghanensis *	JF965996	–			
2	* Barnardiorum shadini *	JF965994	0.1853	–		
3	*Barnardiorum* sp.	JF965995	0.1654	0.1838	–	
4	*Sarothrogammarusyiiruae* sp. nov.	MK770173	0.2009	0.2224	0.2193	–

**Table 2.** GenBank accession numbers and uncorrected pairwise distances of the 28S partial sequences between species in this text.

	Species	Genbank accession number	1	2	3	4
1	* Comatogammarus ferghanensis *	JF965828	–			
2	* Barnardiorum shadini *	JF965826	0.0369	–		
3	*Barnardiorum* sp.	JF965827	0.0497	0.0466	–	
4	*Sarothrogammarusyiiruae* sp. nov.	MK770174	0.0347	0.0395	0.0436	–

## Taxonomy

### Family Gammaridae Leach, 1814

#### Genus *Sarothrogammarus* Martynov, 1935

**Type species.***Sarothrogammarusasiaticus* Martynov, 1935

##### Sarothrogammarus yiiruae

Taxon classificationAnimaliaAmphipodaGammaridae

Hou & Li sp. nov.

http://zoobank.org/6218CB7A-B50B-430C-9F8D-5AC2D3764082

Figs 1 , 2 , 3 , 4 , 5 , 6 , 7 , 8

###### Material examined.

***Holotype*** ♂, 8.0 mm; CHINA, Xinjiang Uygur Autonomous Region, Kizilsu Kirghiz Autonomous Prefecture, Wuqia County, Jigen Town; 39.82N, 74.10E; 2729 m a.s.l.; 26 July 2014; K Meng, YC Lin leg.; IZCAS-I-A1636-1.

***Paratype.*** 1♀, 7.0 mm; same data as for preceding; IZCAS-I-A1636-2.

###### Other materials.

Four males, two females, and two juveniles, same data as for preceding. Two males were used for molecular analysis, but no variation was found between them. Sequences were submitted to GenBank (MK770173 for COI and MK770174 for 28S).

###### Diagnosis.

Eyes absent; antenna II calceoli absent; gnathopods I–II without mid-palmar spine; coxal plate IV weakly concave; bases of pereopods V–VII narrow; uropod I normal, without basofacial spine; uropod III longer than uropods I–II, peduncle about 1/3 length of outer ramus, inner ramus scale-like, with one spine on distal margin, outer ramus biarticulate, first article with four groups of spines on both margins, second article distinct.

**Figure 1.**
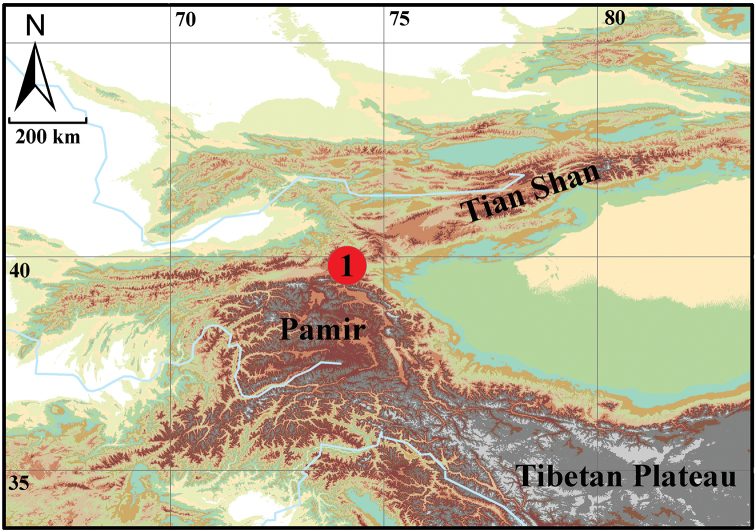
Locality of *Sarothrogammarusyiiruae* sp. nov. in Xinjiang, China.

**Figure 2.**
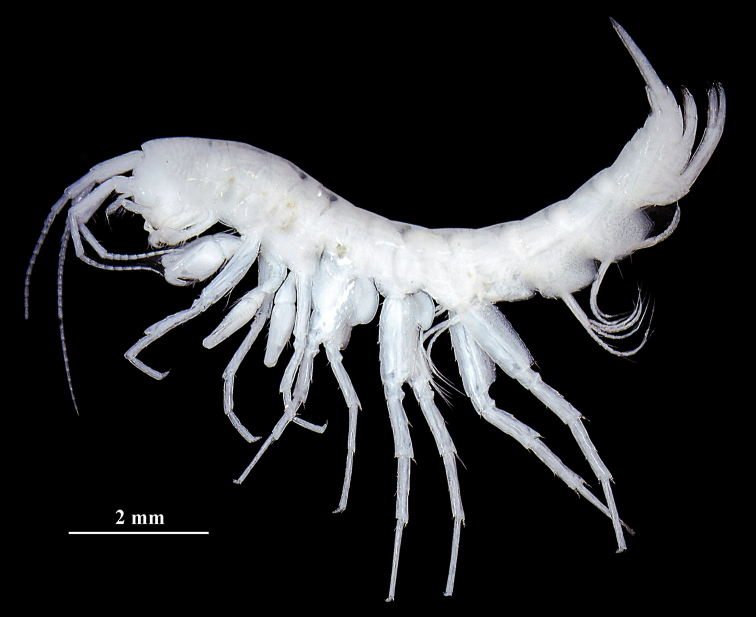
*Sarothrogammarusyiiruae* sp. nov. from Xinjiang, China. Photo showing habitus of male holotype (8.0 mm).

###### Description of male holotype

(IZCAS-I-A1636-1), 8.0 mm.

**Figure 3.**
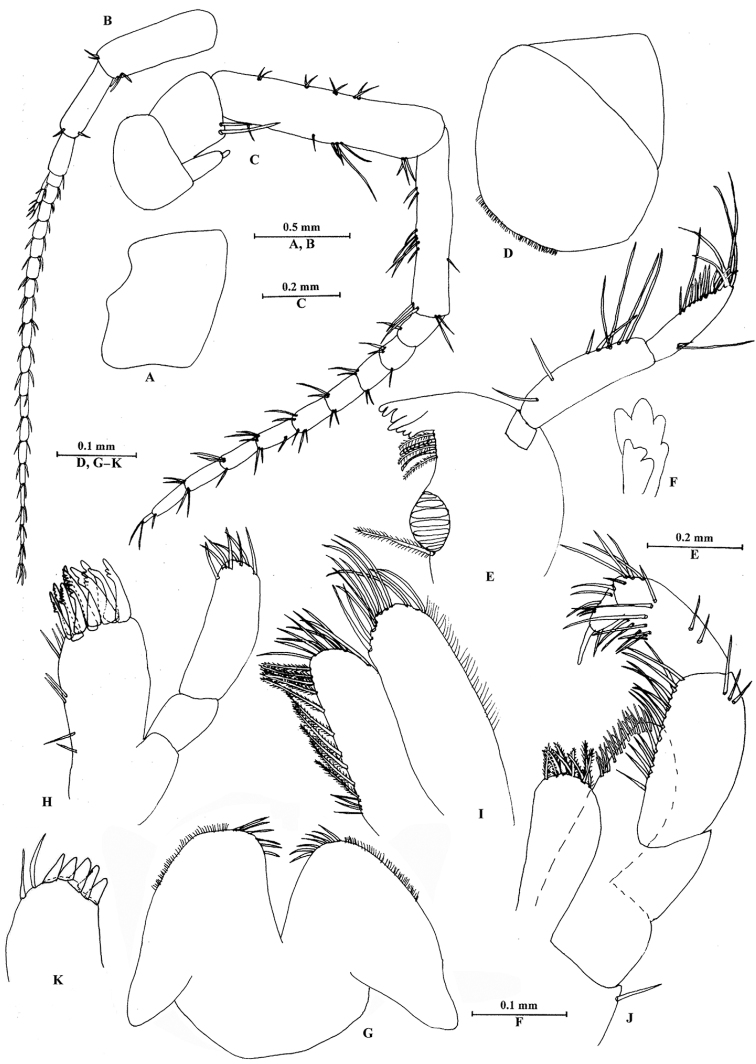
*Sarothrogammarusyiiruae* sp. nov. male holotype, from Xinjiang, China. **A** Head **B** antenna I **C** antenna II **D** upper lip **E** right mandible **F** incisor of left mandible **G** lower lip **H** maxilla I **I** maxilla II **J** maxilliped **K** right palp of maxilla I.

***Head*** (Fig. 3A): eyes absent.

Antenna I (Fig. 3B): longer than antenna II. Peduncle ratio of articles I–III 1.0: 0.6: 0.3, with distal setae; flagellum with 20 articles; accessory flagellum with three articles; both primary and accessory flagellum with short distal setae.

Antenna II (Fig. 3C): peduncle ratio of articles III–V 1.0: 3.0: 2.2, article III with two distal setae, article IV slightly longer than article V, both with setae along anterior and posterior margins; flagellum with ten articles, each article with distal setae; calceoli absent.

Upper lip (Fig. 3D): ventral margin rounded, bearing minute setae.

Mandible (Fig. 3E, F): asymmetrical. Right mandible incisor with four teeth; lacinia mobilis bifurcate; palp composed of three articles, second article with eight setae on inner margin, third article with two B-setae on outer margin, four E-setae on apical margin. Incisor of left mandible with four teeth, lacinia mobilis with three teeth.

Lower lip (Fig. 3G): inner lobe lacking, covered with thin setae.

Maxilla I (Fig. 3H, K): outer plate with nine apical spines, including simple (naked) spines, and spines bearing one, two or multiple dentitions; left palp biarticulate, distal article with eight stiff setae. Distal article of right palp with five stout spines and two setae.

Maxilla II (Fig. 3I): inner plate with plumose and four simple setae medially, nine simple setae apically; outer plate with 12 simple setae apically.

Maxilliped (Fig. 3J): inner plate with nine plumose setae apically; outer plate with spines and setae; palp with four articles, terminal article hooked.

**Figure 4.**
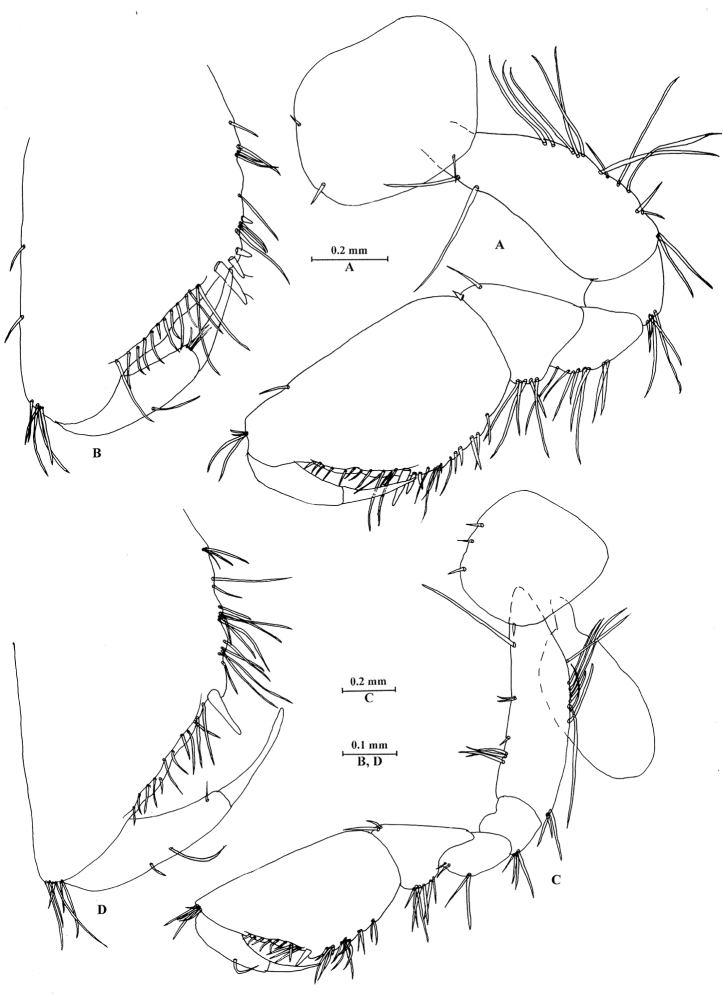
*Sarothrogammarusyiiruae* sp. nov. male holotype, from Xinjiang, China. **A** Gnathopod I **B** propodus of gnathopod I **C** gnathopod II **D** propodus of gnathopod II.

***Pereon.*** Gnathopod I (Fig. 4A, B): coxal plate with two setae on anterior margin, one seta on lower margin; basis sub-linear, with two long setae on anterior margin, 13 unequal setae on posterior margin; merus with eight long setae on posterior margin; carpus with five long setae on posterior margin, tapered distolateral lobe; propodus 1.78 times as long as wide, palmar margin crenellated only in its proximal (angular) part, with 13 setae and defined by three stout spines; dactylus reaching approx. 68% length of propodus, posterior margin arc-shaped, with one seta.

Gnathopod II (Fig. 4C, D): coxal plate with three setae on anterior margin, one seta on lower margin; basis with one long seta and six setae on anterior margin, posterior margin with a row of long setae; merus with two long setae and two short setae on posterior margin; carpus with long setae on posterior margin; propodus 1.8 times as long as wide, palmar margin crenellated only in its proximal (angular) part, with one stout spine and 12 setae, posterior margin with a row of setae extending on proximolateral margin; dactylus reaching approx. 71% length of propodus.

**Figure 5.**
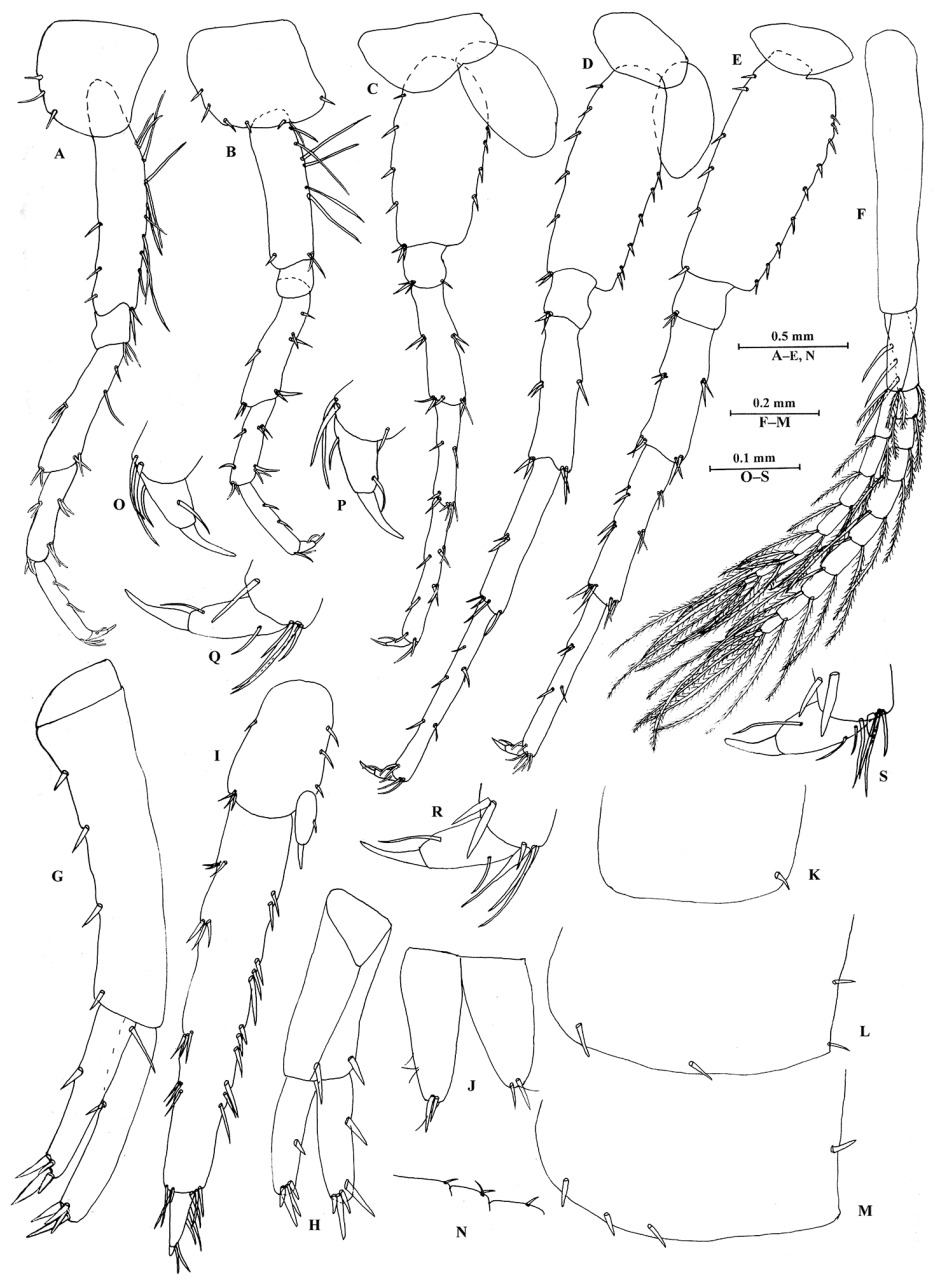
*Sarothrogammarusyiiruae* sp. nov. male holotype, from Xinjiang, China. **A** Pereopod III **B** pereopod IV **C** pereopod V **D** pereopod VI **E** pereopod VII **F** pleopod I **G** uropod I **H** uropod II **I** uropod III **J** telson **K** epimeral plate I **L** epimeral plate II **M** epimeral plate III **N** urosomites (dorsal view) **O** dactylus of pereopod III **P** dactylus of pereopod IV **Q** dactylus of pereopod V **R** dactylus of pereopod VI **S** dactylus of pereopod VII.

Pereopods III–IV (Fig. 5A, B, O, P): similar to each other, pereopod III slightly longer than pereopod IV. Coxal plate III with three setae on anterior margin; coxal plate IV weakly concave, with two setae on anterior margin, one seta on posterior margin and three distal setae; bases sub-linear, bearing unequal spines on both margins; merus to carpus with spines but without long setae on both margins; dactyli with one seta at hinge of unguis.

Pereopods V–VII (Fig. 5C–E, Q–S): similar in shape, pereopod V shorter than pereopods VI and VII. Coxal plates V–VII small, without any spine and setae on both margins; bases narrow, with spines on anterior and posterior margins; dactyli with one seta at hinge of unguis.

Coxal gills present on gnathopod II and pereopods III–VI.

***Pleon.*** Epimeral plates (Fig. 5K–M): plate I ventrally rounded, with one seta on posterior margin; plate II posterior corner blunt, with two setae on ventral margin and two setae on posterior margin; plate III with three setae on ventral margin and one seta on posterior margin.

Pleopods I–III (Fig. 5F): similar to each other. Both rami subequal, with plumose setae.

***Urosome.*** Urosomites I–III (Fig. 5N): with one, two, and one seta on dorsodistal margins, respectively.

Uropod I (Fig. 5G): normal, without basofacial spine; inner ramus slightly shorter than outer ramus, bearing one spine on inner margin; outer ramus with one spine on inner margin; both rami with five terminal spines. Uropod II (Fig. 5H): smaller than uropod I, peduncle with one spine on inner and outer margins, respectively; inner ramus with one spine on outer margin; outer ramus with one spine on outer margin; both rami with four terminal spines. Uropod III (Fig. 5I): longer than uropods I and II, peduncle about 1/3 the length of outer ramus, with one spine on anterior margin, three spines on posterior margin and three spines on distal margin; inner ramus reduced, scale-like, with one spine on distal margin; outer ramus with two articles, first article with four groups of spines on both margins, second article distinct.

Telson (Fig. 5J): deeply cleft, each lobe with two distal spines accompanied by setae.

###### Description of paratype female

(IZCAS-I-A1636-2), 7.0 mm.

**Figure 6.**
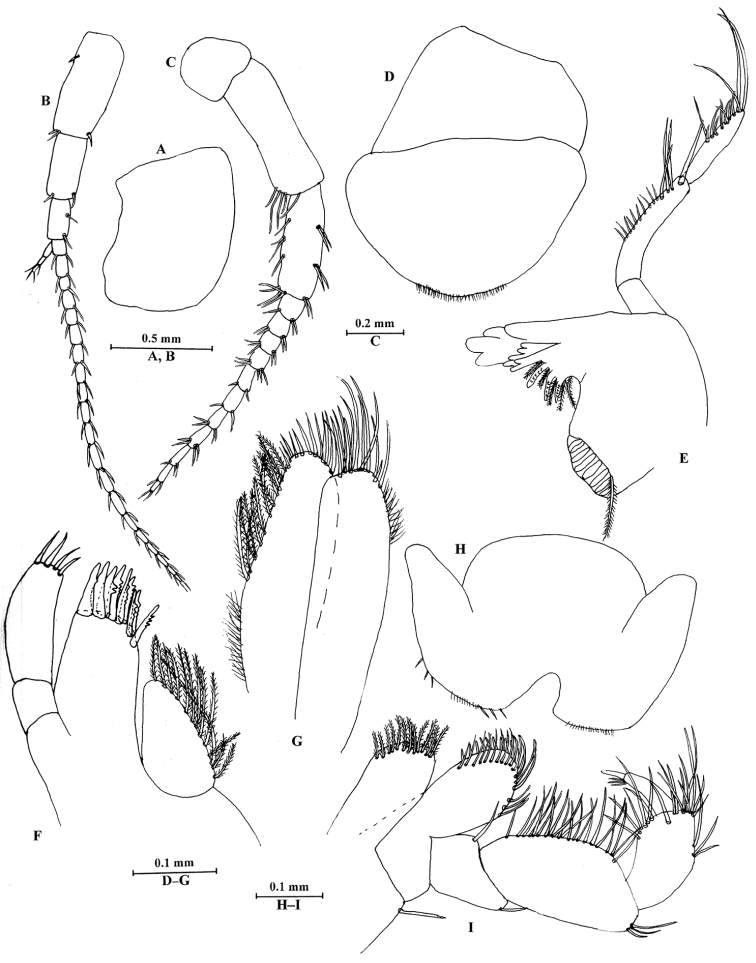
*Sarothrogammarusyiiruae* sp. nov. female paratype, from Xinjiang, China. **A** Head **B** antenna I **C** antenna II **D** upper lip **E** right mandible **F** maxilla I **G** maxilla II **H** lower lip **I** maxilliped.

***Head.*** Antennae and mouthparts (Fig. 6A–I) similar to those of male.

**Figure 7.**
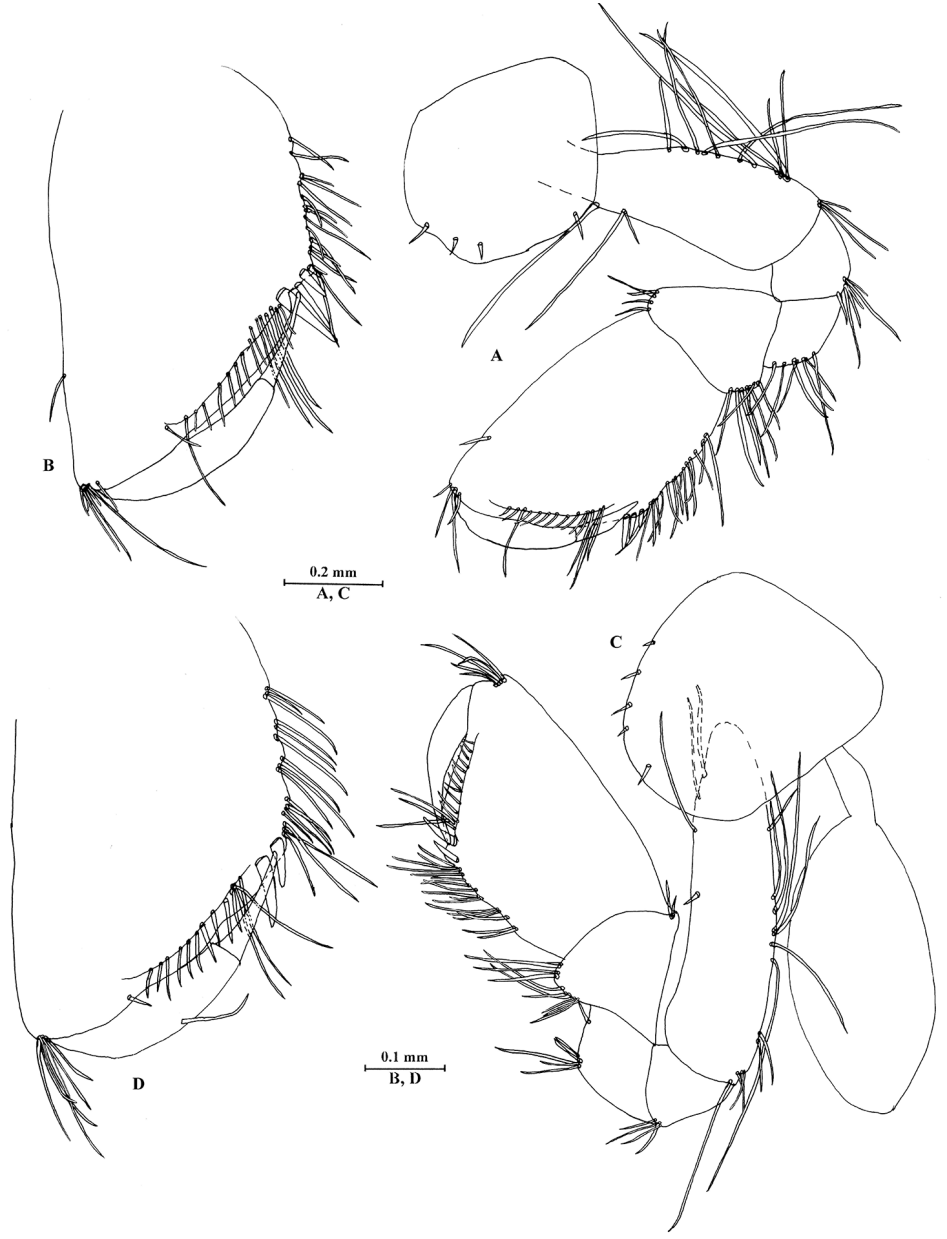
*Sarothrogammarusyiiruae* sp. nov. female paratype, from Xinjiang, China. **A** Gnathopod I **B** propodus of gnathopod I **C** gnathopod II **D** propodus of gnathopod II.

***Pereon.*** Gnathopod I (Fig. 7A, B): Coxal plate with three setae on anterior margin, one seta on distal margin; basis sub-linear; carpus with tapered projection; propodus 1.53 times as long as wide, palmar margin crenellated only in its proximal (angular) part, with four stout spines and 16 setae, posterior margin with a row of setae extending on proximolateral margin; dactylus reaching approx. 68% length of propodus.

Gnathopod II (Fig. 7C, D): Coxal plate with four setae on anterior margin and one distal seta; propodus 1.56 times as long as wide, palmar margin crenellated only in its proximal (angular) part, with two stout spines and 14 setae, posterior margin with a row of setae extending on proximolateral margin; dactylus reaching approx. 67% length of propodus, with one seta on posterior margin.

**Figure 8.**
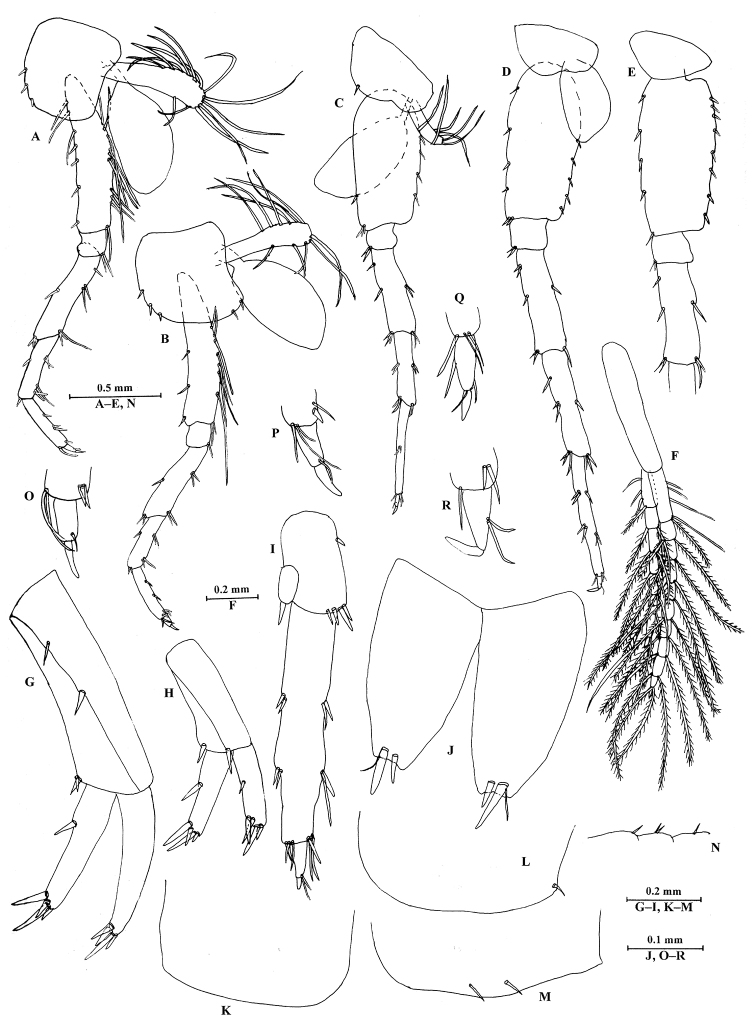
*Sarothrogammarusyiiruae* sp. nov. female paratype, from Xinjiang, China. **A** Pereopod III **B** pereopod IV **C** pereopod V **D** pereopod VI **E** pereopod VII **F** pleopod I **G** uropod I **H** uropod II **I** uropod III **J** telson **K** epimeral plate I **L** epimeral plate II **M** epimeral plate III **N** urosomites (dorsal view) **O** dactylus of pereopod III **P** dactylus of pereopod IV **Q** dactylus of pereopod V **R** dactylus of pereopod VI.

***Pleon.*** Epimeral plates I–III (Fig. 8K–M): plates I–III with no, one and two setae on ventral margins, respectively.

***Urosome.*** Uropod I (Fig. 8G): normal, without basofacial spine; inner ramus with one spine on inner margin; both rami with five terminal spines. Uropod II (Fig. 8H): peduncle with one spine on inner and outer margins, respectively; both rami with four terminal spines. Uropod III (Fig. 8I): peduncle shorter than outer ramus; inner ramus reduced, scale-like, with one spine on distal margin; outer ramus with two articles, first article with two groups of spines on both margins, second article distinct.

###### Etymology.

The specific name is named in honor of Ms Menghe Yiiru, lovely daughter of the collector Meng Kaibayier, for their kind support of Amphipoda research in Xinjiang; noun (name) in genitive case.

###### Habitat.

This species was collected from a stream, rising in snow-capped mountains.

###### Remarks.

The new species is assigned to the genus *Sarothrogammarus* according to the scale-like inner ramus of uropod III and the narrow bases of pereopods V–VII. It is not a member of the genus *Comatogammarus* because pereopod V lacks filtrative setae. It is not assigned to the genus *Barnardiorum* because the second article of the outer ramus in uropod III is distinct rather than vestigial.

*Sarothrogammarusyiiruae* sp. nov. is most similar to *S.trichiatus* Stock, 1971 in having urosomites I–III with setae on the dorsal margin and the shape of uropods I and II. *Sarothrogammarusyiiruae* sp. nov. differs from *S.trichiatus* (character states for *S.trichiatus* in parentheses) in the following: the absence of eyes (the presence of eyes); gnathopod II without mid-palmar spine (with mid-palmar spine); pereopod III without filtrative long setae on merus and carpus (pereopod III with filtrative setae); second article of outer ramus in uropod III distinct, longer than adjacent spines (rudimentary, shorter than adjacent spines). The comparison between the species of sarothrogammarids from the Pamir region is presented in the following key.

We downloaded COI and 28S sequences of sarothrogammarids from Pamir (Hou et al. 2014), including *Comatogammarusferghanensis* (voucher number SLOCHN266), *Barnardiorumshadini* (voucher number SLOCHN263), and *Barnardiorum* sp. (voucher numbers SLOCHN265, 264, 267). Molecular analyses showed high interspecific divergence (Tables 1, 2). The uncorrected distances between *S.yiiruae* sp. nov. and its congeners are higher than 20% for COI, which is larger than the accepted threshold (16%) for crustacean species delimitation (Lefébure et al. 2006). The genetic distances between the new species and its congeners are 3.5–4.4% for 28S, which is comparable to interspecific differentiation for amphipods (Hou et al. 2014). Therefore, morphological and molecular data support *S.yiiruae* sp. nov. being a new species.

### Key to the species of sarothrogammarids from the Pamir region

**Table:** 

1	Pereopod IV with filtrative setae	*** Comatogammarus ferghanensis ***
–	Pereopod IV with no filtrative setae	**2**
2	Second article of outer ramus in uropod III vestigial	**3**
–	Second article of outer ramus in uropod III distinct	**4**
3	Outer ramus of uropod III slender	*** Barnardiorum ruffoi ***
–	Outer ramus of uropod III extended	*** Barnardiorum shadini ***
4	Eyes absent	***Sarothrogammarusyiiruae* sp. nov.**
–	Eyes present	**5**
5	Antenna I peduncle with long setae on ventral margin	*** S. trichiatus ***
–	Antenna I peduncle with short setae on ventral margin	**6**
6	Antenna I accessory flagellum with three or four segments	**7**
–	Antenna I accessory flagellum with two segments	**8**
7	Both male and female pereopod III with rows of long setae	*** S. multipennatus ***
–	Female pereopod III without long setae	*** S. lindbergi ***
8	Gnathopod II palm very oblique	*** S. afghanus ***
–	Gnathopod II palm not oblique	*** S. asiaticus ***

## Supplementary Material

XML Treatment for Sarothrogammarus yiiruae
